# Healthcare resource use, costs and health-related quality of life in the UK–Irish Atopic eczema Systemic Therapy Register (A-STAR): a pilot study

**DOI:** 10.1093/skinhd/vzag042

**Published:** 2026-06-15

**Authors:** Carlos Chivardi, Elizaveta Gribaleva, Bolaji Coker, Man Fung Tsoi, Rebecca Carroll, David Prieto Merino, Manisha Baden, Paula E Beattie, Sara J Brown, Tim Burton, Ross Hearn, John R Ingram, Alan D Irvine, Graham A Johnston, Irene Man, Graham Ogg, Mandy Wan, Richard B Warren, Richard T Woolf, Nick J Reynolds, Michael R Ardern-Jones, Carsten Flohr, Andrea Manca, the UK–Irish A-STAR Group, Mahmud Ali, Michael R Ardern-Jones, Jerome Baldago, Tracy Bale, Paula E Beattie, Sara J Brown, Victoria Brown, Michael J Cork, Ser-Ling Chua, Sharmela Darné, Caoimhe Fahy, Leila Ferguson, Carsten Flohr, Ross Hearn, Nicola Housam, John R Ingram, Graham A Johnston, Effi Ladoyanni, Alison Layton, Claire Leitch, Rosalind Simpson, Irene Man, Deborah Moffit, Louise Newell, Joanne Searle, Sophia Paget, Gabriela Petrof, Urvi Popli, Catherine Rennie, Nick J Reynolds, Nasim Rouhani, Ann Sergeant, Ben Thompson, Shyamal Wahie, Richard B Warren, Richard Weller, Aaron Wernham, Richard T Woolf

**Affiliations:** Centre for Health Economics, University of York, York, UK; A-STAR Register Coordinating Centre, St John’s Institute of Dermatology, Guy’s and St Thomas’ NHS Foundation Trust and King’s College London, London, UK; Research and Development Department, Guy’s and St Thomas’ NHS Foundation Trust, London, UK; A-STAR Register Coordinating Centre, St John’s Institute of Dermatology, Guy’s and St Thomas’ NHS Foundation Trust and King’s College London, London, UK; A-STAR Register Coordinating Centre, St John’s Institute of Dermatology, Guy’s and St Thomas’ NHS Foundation Trust and King’s College London, London, UK; A-STAR Register Coordinating Centre, St John’s Institute of Dermatology, Guy’s and St Thomas’ NHS Foundation Trust and King’s College London, London, UK; Faculty of Medicine, University of Alcalá, Alcalá de Henares, Spain; A-STAR Register Coordinating Centre, St John’s Institute of Dermatology, Guy’s and St Thomas’ NHS Foundation Trust and King’s College London, London, UK; Department of Dermatology, Royal Hospital for Children, NHS Greater Glasgow and Clyde, Glasgow, UK; Centre for Genomic and Experimental Medicine, University of Edinburgh, Edinburgh, UK; Patient Representative (independent), Nottingham, UK; Department of Dermatology and Photobiology, Ninewells Hospital, Dundee, UK; Department of Dermatology, Division of Infection and Immunity, Cardiff University, Cardiff, UK; Department of Clinical Medicine, Trinity College Dublin, Dublin, Ireland; Department of Dermatology, University Hospitals of Leicester NHS Trust, Leicester, UK; Department of Dermatology, Surrey and Sussex Healthcare NHS Trust, Surrey, UK; MRC Translational Immune Discovery Unit, MRC Weatherall Institute of Molecular Medicine, University of Oxford, Oxford, UK; Evelina London Children’s Hospital, Guys’ and St Thomas’ NHS Foundation Trust, London, UK; Institute of Pharmaceutical Science, King’s College London, London, UK; Division of Musculoskeletal and Dermatological Sciences, School of Biological Sciences, Faculty of Biology, Medicine and Health, The University of Manchester, Manchester, UK; Salford Royal Hospital, Northern Care Alliance NHS Foundation Trust, Manchester Academic Health Science Centre, Manchester, UK; St John’s Institute of Dermatology, School of Basic & Medical Biosciences, Faculty of Life Sciences & Medicine, Guy’s and St Thomas’ NHS Foundation Trust, London, UK; Institute of Translational and Clinical Medicine, Newcastle University Medical School and Department of Dermatology and the Newcastle NIHR Biomedical Research Centre, Royal Victoria Infirmary, Newcastle Hospitals NHS Foundation Trust, Newcastle upon Tyne, UK; Faculty of Medicine, University of Southampton, Southampton General Hospital, Southampton, UK; A-STAR Register Coordinating Centre, St John’s Institute of Dermatology, Guy’s and St Thomas’ NHS Foundation Trust and King’s College London, London, UK; Centre for Health Economics, University of York, York, UK

## Abstract

**Background:**

Managing patients with moderate-to-severe atopic eczema (AE) is challenging. Novel systemic immunomodulatory therapies are effective but costly, whereas conventional treatments require more intense safety monitoring. While available randomized trials assess efficacy, they do not reflect real-world practice or cost-effectiveness. The UK–Irish Atopic Eczema Systemic Therapy Register (A-STAR) was established to generate real-world evidence on systemic AE treatments.

**Objectives:**

To assess healthcare resource utilization, costs and health-related quality of life (HRQoL) over 1 year in participants in A-STAR, and to evaluate data quality in a pilot analysis.

**Methods:**

A-STAR is a multicentre prospective register that recruits paediatric (aged <16 years) and adult (aged ≥16 years) patients with AE who are initiating or switching systemic immunomodulatory therapy. Healthcare utilization [general practitioner (GP) visits, accident and emergency (A&E) department attendance, hospitalizations, specialist consultations and therapy costs] was valued using national average unit costs and tariffs. HRQoL was measured with the EuroQol 5 Dimension (EQ-5D).

**Results:**

Of 120 participants (92 adults and 28 children) with a median follow-up to 12 months, adults had higher mean healthcare costs per year than children, including A&E (£120.41 vs. £84.29), GP (£111.15 vs. £78.46) and specialist (£205.17 vs. £121.00) visits. Mean (SD) systemic therapy costs per year were £25 523 (£24 424) in adults and £20 242 (£18 994) in children, averaged across all treatment options. Mean EQ-5D scores improved from baseline to 1 year (from 0.608 to 0.769 in adults and from 0.482 to 0.751 in children).

**Conclusions:**

Systemic therapy improved HRQoL but incurred notable costs. A-STAR is well positioned to support future comparative economic evaluations of alternative treatment strategies to inform clinical and reimbursement decisions.

## Teaser

Managing patients with moderate-to-severe atopic eczema (AE) involves balancing the high cost of novel therapies with the monitoring demands of conventional treatments, but real-world data are limited. This pilot study from the Atopic eczema Systemic Therapy Register (A-STAR) registry assessed healthcare resource use, costs and health-related quality of life (HRQoL) over 1 year in the first 120 patients with AE starting or switching systemic therapy. The results showed significant treatment costs and improved HRQoL during treatment, highlighting the value of A-STAR for informing future cost-effectiveness and safety comparisons.

What is already known about this topic?

- Atopic eczema (AE), also known as atopic dermatitis, is a chronic skin condition that often begins in early childhood and can persist into adulthood.
- Moderate-to-severe AE places a significant economic burden on patients, their families and society at large.
- AE affects patients’ health-related quality of life (HRQoL), in turn affecting their physical, emotional and social wellbeing.
- There is a lack of long-term comparative real-world data on the (cost-)effectiveness and safety of systemic AE treatments from large-scale multicentre studies.

What does this study add?

- This study reports real-world evidence on the use of systemic immunomodulatory therapies for AE through the UK–Irish Atopic Eczema Systemic Therapy Register (A-STAR).
- We provide information on the healthcare resource utilization, costs and HRQoL reported by children and adults with ­moderate-to-severe AE during the first year of entering the register.
- We demonstrate the feasibility of capturing good-quality real-world data on the cost and HRQoL reported by patients with ­moderate-to-severe AE receiving systemic therapy.

Atopic eczema (AE), also known as atopic dermatitis, is a chronic skin condition characterized by itchy, dry and cracked skin. It often manifests before a child’s first birthday, although it can also develop *de novo* in adults.^1^ Approximately 30% of cases of early AE persist into adulthood.^2^

The prevalence of AE has increased two- to threefold over the last century. It now affects up to 20% of children and 4–7% of adults in the UK and other European countries.^2,3^ In adults, ­moderate-to-severe AE has been estimated to result in annual societal costs across Europe of approximately €30 billion; half of this can be attributed to missed workdays or reduced work productivity, while two-thirds of the remaining half are direct medical costs. The rest is related to personal costs. In addition, AE can substantially affect a patient’s physical, emotional and social wellbeing, affecting their overall quality of life.^4^

Most children and adults with mild AE can be treated effectively with emollients and topical anti-inflammatory agents. However, around 5% of patients require systemic immunomodulatory therapies.^5^ Currently, ciclosporin is the only conventional systemic treatment approved in Europe for patients aged >16 years of age with AE.^6^ Its long-term use is limited, in particular due to the risk of renal toxicity and hypertension. Alternative conventional systemic immunomodulatory therapies include methotrexate and azathioprine.^2^ Biologic and small-molecule treatments (e.g. dupilumab, tralokinumab, lebrikizumab, abrocitinib, baricitinib and upadaci­tinib) are also approved for the treatment of moderate-to-severe AE, but in the UK National Health Service (NHS) these therapies can only be used as second-line therapies (or in those for whom conventional systemics are contraindicated), as per National Institute for Health and Care Excellence (NICE) recommendations.^7^

To date, there is no long-term comparative real-world evidence of the costs, effectiveness and safety of the available systemic immunomodulatory treatments for AE in children and adults from large-scale multicentre studies.^8–10^ This information gap results in a lack of clear management guidance to inform clinical practice, which was echoed by the Multiple Technology Appraisal, conducted by NICE in 2022,^11^ comparing abrocitinib, tralokinumab and upadacitinib in the treatment of moderate-to-severe AE. In its report, NICE highlighted the shortcomings of the current evidence base and the important role the UK–Irish Atopic Eczema Systemic TherApy Register (A-STAR) plays in collecting such (cost-)effectiveness and safety data.^12^

Therefore, the setup of a prospective multicentre register study is important to capture the real-world use of systemic immunomodulatory therapies in paediatric and adult patients, and to facilitate the assessment of these therapies in terms of their effectiveness, safety and economic outcomes beyond the confines of short-term selective randomized controlled trials (RCTs).^12^

The UK–Irish A-STAR is a prospective multicentre UK–Irish study designed to meet the above evidentiary needs (https://astar-register.org).^13–15^ Baseline characteristics of the A-STAR cohort have been described recently.^16^ Here, we complement this information by reporting the results of a pilot study, which analysed healthcare resource use, costs and health-related quality of life (HRQoL) in the first 120 participants in A-STAR recruited after the COVID-19 pandemic, with 12 months of follow-up. The objectives were to (i) provide an expert review of the information being collected; (ii) discuss the areas of strength and weakness of the health economics data available in the register; (iii) facilitate considerations of alternative data-collection strategies for this information; and (iv) highlight the potential uses of A-STAR health economics data to inform NICE decision-making.

## Materials and methods

### Selection of the population and study design

Full details of the A-STAR cohort design, recruitment procedures and eligibility criteria have been published recently, alongside the baseline characteristics, comorbidities and prior treatment history of participants.^16^ Patients with moderate-to-severe AE initiating or switching to a systemic immunomodulatory agent were eligible for inclusion. Patients were recruited across multiple sites in the UK and Ireland, as part of routine dermatology care. There was no upper or lower age limit for participation. For the purposes of analysis, participants were classified as paediatric (aged <16 years) or adult (aged ≥16 years) patients. The age threshold of 16 years was chosen in accordance with UK clinical practice, where patients aged ≥16 years are typically managed within adult dermatology services, and to reflect age-specific treatment indications and clinical guidelines for systemic therapies in AE. Herein, we describe a comprehensive health resource use and generic quality of life analysis of the first 120 participants in A-STAR recruited after the COVID-19 pandemic, with 12 months of follow-up.

### Health economic data collection

Healthcare resource use data were collected via self-reported questionnaires (electronic case report form; eCRF). Participants were asked to report the number of eczema-related visits to different types of healthcare providers in the previous 12 months.

### Healthcare resource use

We quantified the consumption of each item of healthcare resource per patient at each follow-up visit and aggregated these quantities to estimate the total amount of healthcare resources utilized during follow-up. Items of resource use collected in the register include (among others): accident and emergency (A&E) department attendance; primary care general practitioner (GP) visits; use of systemic therapy; and visits with other healthcare professionals related to AE. Healthcare resource use was valued in monetary terms by applying routinely collected national average unit cost figures relevant to the UK NHS. Key data sources for the unit costs include NHS Reference Costs, British National Formulary drug prices (https://bnf.nice.org.uk/) and the Personal Social Services Research Unit survey of unit costs (https://research.kent.ac.uk/corec/).^17^ Resource use was costed using UK national figures at 2022 prices. More details are provided in Table S1 (see Supporting Information).

### Cost estimation

The cost analysis was conducted from the UK healthcare-payer perspective, meaning that all cost calculations reflect direct medical expenditures borne by the health system. Costs for an individual at each follow-up time were calculated as the sum of the quantity of each healthcare resource used multiplied by its relevant unit cost. Total costs for an individual were derived by integrating item-specific costs at each follow-up interval over the study period. To assess the importance of resource items in terms of their overall contribution to the total cost, data are required on the frequency of consumption.^18^ For each item of resource use, we also reported the proportion of individuals in the sample who had zero consumption of specific items of healthcare resources. The cost estimates reported in this study reflect only the healthcare resources explicitly captured in the eCRF of the A-STAR register, which include systemic therapy use and healthcare contacts (i.e. GP, A&E, outpatient specialist and dermatology visits), valued using national UK unit cost sources. Other potentially relevant cost components, such as laboratory monitoring, imaging or additional investigations associated with specific therapies, were not systematically recorded in the register at this pilot stage and were therefore not included in the analysis. Future amendments of the eCRF will consider the inclusion of more detailed healthcare resource use data.

### Health-related quality of life

HRQoL refers to an individual’s or a population’s perceived physical, mental and social wellbeing in relation to their health status. The EuroQol 5 Dimension (EQ-5D),^19^ a preference-based patient-­reported outcome that measures an individual’s generic HRQoL, was completed by patients at baseline and at each follow-up visit alongside other questionnaires designed to measure clinical outcomes. The EQ-5D is the instrument of choice to measure health benefits for use in economic evaluation studies submitted for consideration to NICE.^20^ Here, we adopted the generic five-­dimension (i.e. mobility, self-care, activities, pain/discomfort and ­anxiety/depression) five-level version (EQ-5D-5L) for adults and the EQ-5D-Y for participants in the paediatric age range.^21^ When responding to the questionnaire, for each of the dimensions, respondents classified their health status between 1 (no problem) and 5 (extreme problems) in the five-level version for adults and between 1 (no problem) and 3 (severe problems) in the three-level version used by children (https://euroqol.org/information-and-support/euroqol-instruments/). Official valuation tariffs for the UK are not yet available for the EQ-5D-5L, so we used the approach recommended by NICE, in that the EQ-5D-5L was mapped onto the EQ-5D-3L scale and ‘scored’ using the UK population norms for the latter. We report the mean EQ-5D index values calculated for the adult and paediatric samples, their SD, 95% nonparametric confidence interval and percentage of missing questionnaires at each follow-up visit. The analysis was performed using R version 4.3.1 (R Foundation for Statistical Computing, Vienna, Austra), package ‘eq5d’, and STATA 18 MP (StataCorp., Cary, NC, USA).

## Results

### Baseline characteristics

The baseline characteristics of the study sample are provided in Table 1. A total of 120 (92 adults and 28 children) participants were included in the analysis. Children had a mean (SD) age of 12.0 (3.4) years, while the adult participants had a mean (SD) age of 37.7 (13.8) years. The sex distribution at birth was similar in both groups. Ethnicity differed between groups. In children, 50% were White, 36% were Asian and 10% were Black African. However, adults were predominantly White (83%), with smaller proportions of people identifying as Asian (10%) and Black African (2%).

**Table 1:** Baseline characteristics of the participants in A-STAR included in this study

Characteristic	Children (*n* = 28)	Adults (*n* = 92)
Age (years), median (IQR)	12 (10–14)	33 (26–49)
POEM, mean (SD)	17.93 (7.20)^a^	20.17 (6.95)^b^
CDLQI, mean (SD)	15.17 (8.01)^c^	15.90 (8.20)^d^
PP-NRS, mean (SD)	6.36 (2.84)^e^	7.11 (2.16)^f^
No. of prior systemic treatments, mean (SD)^g^	2.22 (1.39)^h^	2.61 (1.77)^i^
Sex		
Male	17 (61)	55 (60)
Female	11 (39)	36 (39)
Missing	0 (0)	1 (1)
Food allergy		
No	12 (43)	48 (52)
Yes	15 (54)	42 (46)
Missing	1 (4)	2 (2)
Contact allergies		
No	17 (61)	46 (50)
Yes	5 (18)	34 (37)
Unknown	3 (11)	9 (10)
Missing	3 (11)	3 (3)
Aeroallergen sensitization		
Yes	4 (14)	40 (43)
No	17 (61)	20 (22)
Unknown	2 (7)	27 (29)
Missing	5 (18)	5 (5)
Ethnicity		
White	14 (50)	76 (83)
Asian	10 (36)	9 (10)
Black African	3 (11)	2 (2)
Other^j^	1 (4)	4 (4)
Missing	0 (0)	1 (1)

Data are presented as *n* (%) unless otherwise stated. A-STAR, UK–Irish Atopic Eczema Systemic Therapy Register; CDLQI, Children’s Dermatology Life Quality Index; IQR, interquartile range; POEM, Patient-Oriented Eczema Measure; PP-NRS, Peak Pruritus Numerical Rating Scale. ^a^*n* = 27. ^b^*n* = 89. ^c^*n* = 23. ^d^*n* = 88. ^e^*n* = 22. ^f^*n* = 83. ^g^Number of distinct systemic therapies previously received by each participant before enrolment in A-STAR. ^h^*n* = 18. ^i^*n* = 68. ^j^‘Other’ includes participants identifying as Hispanic or Latino, Black American and those selecting ‘Other’ with free-text specification.

### Resources use utilization

Table 2 shows the frequency of visits [A&E, GP, outpatient specialist care (excluding dermatology) and dermatology outpatient appointments] during the study period. In the paediatric cohort, there was minimal use of A&E, GP and specialist services throughout the year, with consistently low rates (close to 0%) for most of the year across these services. Dermatology, initially used by all the children, showed a substantial drop, ending the year with 86% use. For adults, the pattern of low use was similar for A&E, GP and specialist services, but slightly more variable than found in the paediatric group. In contrast, dermatology outpatient visits were initially high and remained the most-used service among adults throughout the year, although it also exhibited some decline, stabilizing at around 75–90% use by the end of the year (Table 2).

**Table 2:** Healthcare resource use in A-STAR over the initial 12-month follow-up

Visit	A&E	GP	Specialist outpatient^a^	Dermatology
Children (*n* = 28)				
Baseline				28 (100)
1 months	0 (0)	1 (4)	0 (0)	12 (43)
3 months	1 (4)	1 (4)	1 (4)	26 (93)
6 months	1 (4)	0 (0)	2 (7)	14 (50)
9 months	0 (0)	0 (0)	1 (4)	24 (86)
1 year	0 (0)	1 (4)	1 (4)	24 (86)
Adults (*n* = 92)				
Baseline				90 (98)
1 months	2 (2)	3 (3)	6 (7)	26 (28)
3 months	2 (2)	5 (5)	7 (8)	83 (90)
6 months	1 (1)	6 (7)	4 (4)	69 (75)
9 months	2 (2)	12 (13)	3 (3)	71 (77)
1 year	3 (3)	5 (5)	6 (7)	80 (87)

Data are presented as *n* (%). A&E, accident and emergency department; A-STAR, UK–Irish Atopic Eczema Systemic Therapy Register; GP, general practitioner. ^a^Excluding dermatology.

**Figure 1:**
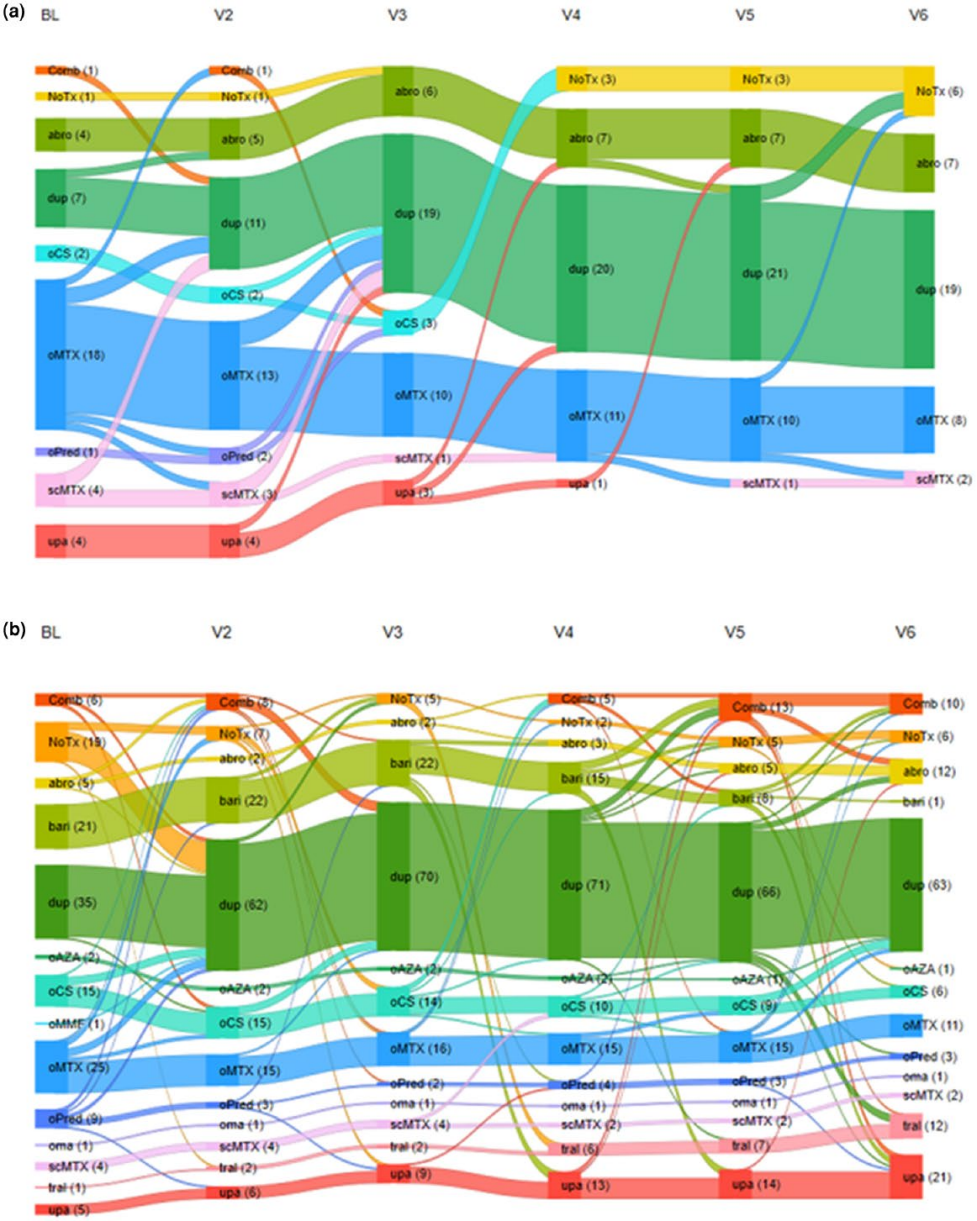
Sankey diagram representing the systemic therapies received in (a) children and (b) adults. Some patients received combination therapies at one or more timepoints, which is why the total number of treatments exceeds the sample size. The diagram represents the total number of treatments per visit and does not correspond to individual patient trajectories. Some patients experienced delays in treatment initiation, which is reflected as having period with no systemic treatment ongoing. abro, abrocitinib; bari, baricitinib; BL, baseline; Comb, combination of several treatments; dup, dupilumab; NoTx, no systemic therapy; oAZA, oral azathioprine; oCS, oral ciclosporin; oma, omalizumab; oMMF, oral mycophenolate mofetil; oMTX, oral methotrexate; oPred, prednisolone; scMTX, subcutaneous methotrexate; tral, tralokinumab; upa, upadacitinib; V, visit.

Figure 1 illustrates the number, range and sequence of systemic therapies administered at each timepoint over six study visits (from baseline to visit 6) for children and adults. In Figure 1, each vertical block represents the total number of systemic therapies recorded at a given visit, while the connecting flows represent transitions between therapies across consecutive visits. The width of each flow is proportional to the number of therapies administered across the sample, allowing visual assessment of treatment stability and switching patterns over time. Some individuals were reported to be receiving multiple therapies at each visit, which is why the total number of treatments does not correspond to the sample size in each group.

Treatment patterns among children were relatively stable, with fewer transitions between therapies, which may be due, in part, to the limited number of therapies licensed for use in the paediatric population vs. the adult population (Figure 1a). Several treatments, particularly dupilumab and oral methotrexate, remained prominent across visits. The increasing continuity of dupilumab over time suggests sustained treatment use in this group, while other therapies appeared intermittently, reflecting changes driven by patient needs, treatment effectiveness or safety considerations.

In contrast, there is a more complex pattern of treatment use in adults, characterized by a wider range of therapies and more frequent switching (Figure 1b). Dupilumab remained a consistently used treatment across visits, alongside notable use of oral ciclosporin and oral methotrexate, highlighting the greater therapeutic heterogeneity and dynamic treatment strategies in adult patients.

### Cost results

Table 3 presents the cost analysis for the paediatric group. The majority of children (*n* = 26/28; 93%) did not report any A&E attendances during the study period. The mean cost per A&E attendance, when these occurred, was £84.20. However, when considering the entire sample (*n* = 28), including those without any A&E visits, the mean (SD) cost of A&E admissions dropped to £6.00 (£22.10). The cohort reported low use of GP appointments, with 89% of the children (*n* = 25/28) indicating not having seen a GP during the first 12 months from registration. The mean cost per GP visit for the three participants who reported a nonzero use was £78.40. As before, when considering the entire paediatric sample, the mean (SD) cost decreased to £8.40 (£30.80). Only five children in this cohort reported having had at least one additional out­patient specialist visit during the study time. The mean cost per outpatient specialist visit estimated from nonzero values was £121.00. However, when considering all participants, the mean (SD) cost dropped to £21.60 (£47.10). All participants reported having received at least one systemic therapy. Systemic therapy represented a substantial component of the overall costs, with a mean (SD) annual cost of £20 849 (£18 994) per child across all systemic therapies combined. All children had costs associated with dermatology visits [mean (SD) £777.10 (£156.40)].

**Table 3:** Cost by category of resource use in A-STAR over the initial 12 months of follow-up

Category	Zero utilization, *n* (%)	Mean cost (£)^a^	Mean (SD) cost (£)^b^	95% CI^c^
Children (*n* = 28)				
A&E visit	26 (92.86)	84.29	6.02 (22.10)	0.00–84.29
GP visit	25 (89.29)	78.46	8.40 (30.86)	0.00–156.92
Specialist visit^d^	23 (82.14)	121.00	21.60 (47.19)	0.00–121.00
Dermatology visit	0	NA	777.14 (156.41)	510.00–1020.00
Systemic therapy	0	NA	20 242.00 (18 994.00)	15.64–61 972.00
Adults (*n* = 92)				
A&E visit	85 (92.39)	120.41	9.16 (35.21)	0.00–168.58
GP visit	74 (80.43)	111.15	21.74 (63.00)	0.00–183.40
Specialist visit^d^	69 (75.00)	205.17	51.29 (107.97)	0.00–363.00
Dermatology visit	0	NA	551.00 (112.54)	281.32–726.00
Systemic therapy	0	NA	25 342.00 (24 424.00)	13.31–55 530.00

A&E, accident and emergency department; A-STAR, UK–Irish Atopic Eczema Systemic Therapy Register; CI, confidence interval; GP, general practitioner; NA, not applicable. ^a^Mean cost among those who reported having used healthcare resources. ^b^Sample statistics, which includes a zero cost for those who required no healthcare contact. ^c^Nonparametric, based on 2.5th and 97.5th centiles of the empirical distribution. ^d^Excluding dermatology.

Most adult participants (*n* = 85/92; 92%) did not attend A&E in the first 12 months of observation. The mean cost per A&E attendance, calculated from those who reported this event, was £120.40. However, because most participants reported no A&E events, the mean (SD) cost for the entire adult sample decreased to £9.80 (£35.20). GP visits followed a similar pattern, with 20% of adults (*n* = 18/92) attending at least one GP visit during the study period. The mean cost per GP visit, among those reporting having had primary care visits, was £111.10. However, in the entire adult sample, the mean (SD) cost of GP visits was only £21.70 (£63.00). Additional specialist visits were the most frequently reported, with 25% of adults (*n* = 23/92) having at least one such visit with a mean cost, for those who required them, of £205.10. When including the 75% of adults (*n* = 69/72) who required no additional specialist visits, the mean (SD) cost dropped to £51.20 (£107.90). All adults had costs associated with dermatology visits [mean (SD) cost £551.00 (£112.50); range £281.30–£726.00]. Finally, systemic therapy as a class of drugs was a considerable cost driver, as expected, with all adult participants receiving systemic treatment during the study period. The mean (SD) cost of systemic therapy was £25 523 (£25 405), with substantial variability observed.

### Quality of life (EQ-5D) results

Table 4 shows the results of the EQ-5D analysis. At baseline, 83 adults had a mean (SD) score of 0.602 (0.311), while 21 children had a lower mean (SD) score of 0.428 (0.367). By the second visit (4 weeks) the mean (SD) EQ-5D score rose to 0.669 (0.244) for adults and 0.662 (0.340) for children. The third visit (3 months) showed further improvement, with mean (SD) scores of 0.750 (0.287) for adults and 0.729 (0.315), for children. Subsequent visits (6, 9 and 12 months) showed continued improvement, with final mean (SD) scores of 0.764 (0.246) for adults and 0.751 (0.266) for children. Detailed EQ-5D scores trajectories and missing patterns are in Figures S1–S4 and Table S2 (see Supporting Information).

**Table 4:** EQ-5D scores by visit and population

	Adults (*n* = 92)				Children (*n* = 28)			
Visit	*n*	Missing (%)	Mean (SD)	Range	*n*	Missing (%)	Mean (SD)	Range
Baseline	79	14	0.602 (0.311)	−0.266 to 1	21	25	0.482 (0.367)	−0.167 to 1
1 month	30	67	0.669 (0.244)	0.094 to 1	18	36	0.662 (0.340)	−0.189 to 1
3 months	54	41	0.750 (0.287)	0.076 to 1	19	32	0.729 (0.315)	0.076 to 1
6 months	47	49	0.712 (0.320)	−0.239 to 1	16	43	0.751 (0.266)	0.052 to 1
9 months	46	50	0.762 (0.246)	−0.092 to 1	17	39	0.769 (0.299)	0.076 to 1
1 year	55	40	0.764 (0.238)	−0.155 to 1	13	54	0.751 (0.266)	0.155 to 1

EQ-5D, EuroQol 5 Dimension.

## Discussion

This descriptive pilot analysis of the paediatric sample in our cohort showed low utilization of A&E and GP services, reflecting the few children requiring such services. Additional specialist visits were also infrequent. Systemic therapy was a notable expense at a mean cost of £20 849 per child, with a limited range of drugs being used, perhaps reflecting the licensing indication of these products in the paediatric population. In contrast, adults showed a similar pattern of low A&E and GP use, but higher mean costs for specialist visits and systemic therapy (£25 523). The difference in mean treatment cost between children and adults is expected, given that most of the treatments are weight-based and adults weigh more than children. Similarly, the greater variability of systemic therapies used in adults is probably a reflection of a wider range of treatment options available to clinicians in this group. Overall, the average healthcare resource use was low across the adult and paediatric A-STAR cohort. The EQ-5D analysis indicated that, on average, children and adults experienced improvements in their generic HRQoL over the first 12 months of follow-up since registration in A-STAR.

One of the challenges facing A-STAR data at the time of analysis is distinguishing between missing data due to unattended study visits and missing data from completed visits where the information was not recorded or not applicable. This is an important distinction, because in some cases missing data may represent zero occurrences of a visit, but in others it may indicate that the visit occurred, but the information was not reported. A clearer distinction between these cases will be an important improvement in data collection and reporting.

We also found some inconsistency in data acquisition, especially in high-recruiting sites, which did not always collect EQ-5D data systematically. This may have been due to a combination of factors. One reason may be that visit 2 in A-STAR was not the standard of care in some centres, and the EQ-5D is usually not included as an HRQoL assessment tool in routine NHS practice. As the quality and accuracy of the A-STAR data matures over time, with larger sample sizes and longer patient follow-up, we expect to have more informative data, in particular to conduct comparative analyses between systemic treatments.

The findings of this study should be interpreted in the context of the UK and Irish healthcare systems, where dermatology services for moderate-to-severe AE are predominantly delivered in secondary and tertiary care. In healthcare settings where dermatology provision is primarily based in office-based practices, patterns of resource use and associated costs may differ. Nevertheless, the clinical treatment pathways and therapeutic decision-making for systemic therapies are largely comparable across many high-income countries, and the real-world evidence generated by A-STAR provides insights that remain relevant for international audiences.

At present, A-STAR pharmacovigilance reporting relies on site staff gathering adverse event data from patients retrospectively. In the future, we will also include pharmacovigilance data captured through data linkage, for instance the NHS Hospital Episodes Statistics (HES) platform.

The collection of longitudinal real-world evidence offers several key advantages over data from RCTs: it enables the study of disease progression, allows for extended patient follow-up and captures real-world treatment patterns. These data are also essential for evaluating the transportability and generalizability of RCT findings. Treatment registers can more easily adapt their data collection to include new clinical variables (e.g. biomarkers), novel therapies and complex treatment sequences that would be difficult to assess in the context of an RCT. Similarly, longitudinal register data can incorporate important cost elements outside the healthcare payer’s perspective, such as the time patients spend away from their usual activities (e.g. work and school). These cost elements are relevant for Health Technology Assessment (HTA) decisions in some jurisdictions (e.g. Sweden and the Netherlands) but not others. A-STAR has collected this information, and future analyses with longer follow-up and a larger sample size may help clarify the extent to which different systemic therapies can reduce societal costs by enabling an earlier return to normal activities.

An even more comprehensive approach could include linking A-STAR patient-level data with electronic health records routinely collected and managed by NHS England, such as primary data from the Clinical Practice Research Data, or secondary data from HES and mortality from the Office for National Statistics. Whether this linkage would be a cost-effective investment, compared with continuing to rely on direct data collection in A-STAR, depends on the study question for which the data are needed. For HTA-type questions, like those that NICE may be interested in, given that the majority of the healthcare costs found in our sample during the first year are represented by the cost of systemic therapies, the above linkage may not be required.

Our study provides the first detailed report of 1-year follow-up health economics data from the A-STAR register, offering insights into healthcare resource use, associated costs and changes in HRQoL in a real-world setting. Our findings highlight systemic therapies as the major driver of healthcare costs and demonstrate improvements in HRQoL in patients with AE over the first year of treatment.

Our study integrates the information presented in an earlier report,^16^ which described the cohort design, recruitment procedures and eligibility criteria in A-STAR, alongside the cohort’s baseline characteristics, comorbidities and prior treatment history. We complete the picture by reporting longitudinal healthcare resource utilization, cost and HRQoL in a subset of participants in A-STAR during their first year since registration, illustrating the complex dynamics of treatment switching in routine clinical practice. These findings represent a valuable step towards understanding the challenges associated with a realistic evaluation of the effect of systemic drugs in the long term, their economic and health outcomes, and the value of longitudinal registers in benchmarking RCT treatment protocols against real-world clinical ­practice. As A-STAR continues to mature, the register is uniquely positioned to inform cost-effectiveness assessments, guide policy decisions and complement clinical trial data through high-quality real-world evidence that reflects patient journeys in NHS settings more closely than clinical trials.

## Supplementary Material

vzag042_Supplementary_Data

## Data Availability

The data underlying this article will be shared on reasonable request to the A-STAR Chief Investigator, Professor Carsten Flohr (carsten.flohr@kcl.ac.uk).

## Appendix 1 A-STAR Register Study Group (in alphabetical order)

Mahmud Ali (University Hospitals Sussex NHS Foundation Trust), Michael R. Ardern-Jones (University of Southampton), Jerome Baldago (Chelsea and Westminster Hospital NHS Foundation Trust), Tracy Bale (Aneurin Bevan University Health Board), Paula E. Beattie (Royal Children’s Hospital Glasgow), Sara J. Brown (University of Edinburgh), Victoria Brown (West Hertfordshire Hospitals NHS Trust), Michael J. Cork (Sheffield Teaching Hospital and Sheffield Children’s Hospital), Ser-Ling Chua (University Hospitals Birmingham), Sharmela Darné (North Tees and Hartlepool NHS Foundation Trust and The James Cook University Hospital NHS Trust), Caoimhe Fahy (County Durham and Darlington NHS Trust Foundation), Leila Ferguson (St George’s University Hospitals NHS Foundation Trust), Carsten Flohr (Guy’s and St Thomas’ NHS Foundation Trust), Ross Hearn (University of Dundee), Nicola Housam (United Lincolnshire Hospitals NHS Trust), John R. Ingram (University Hospital of Wales), Graham A. Johnston (Leicester Royal Infirmary), Effi Ladoyanni (University Hospitals Birmingham), Alison Layton (Harrogate and District NHS Foundation Trust), Claire Leitch (University of Edinburgh), Rosalind Simpson (Nottingham University Hospitals NHS Trust), Irene Man (Surrey and Sussex Healthcare NHS Trust), Deborah Moffit (Gloucestershire Hospitals NHS Foundation Trust), Louise Newell (Bristol Royal Infirmary and Bristol Children’s Hospital), Joanne Searle (University of Oxford), Sophia Paget (Epsom and St Helier University Hospitals NHS Trust), Gabriela Petrof (Great Ormond Street Hospital for Children), Urvi Popli (Glan Cwyd Hospital), Catherine Rennie (Norfolk and Norwich University Hospital), Nick J. Reynolds (University of Newcastle), Nasim Rouhani (London North West University Healthcare NHS Trust), Ann Sergeant (NHS Fife), Ben Thompson (Liverpool University Hospitals NHS Foundation Trust), Shyamal Wahie (North Tees and Hartlepool NHS Foundation Trust and The James Cook University Hospital NHS Trust), Richard B. Warren (University of Manchester), Richard Weller (University of Edinburgh), Aaron Wernham (Walsall Healthcare NHS Trust), Richard T. Woolf (Guy’s and St Thomas’ NHS Foundation Trust).
