# Supplementary material for: Healthcare resource use, costs and health-related quality of life in the UK–Irish Atopic eczema Systemic Therapy Register (A-STAR): a pilot study
Source: Skin Health Dis. 2026 Jun 15;6(4):459–67. doi: 10.1093/skinhd/vzag042 (PMC13425030; doi:10.1093/skinhd/vzag042)
Supplement: vzag042_Supplementary_Data [file vzag042_supplementary_data.docx]

Supplementary material

***Table A1.*** Unit cost of the inputs used in the analysis.

| Inputs | Resource unit | Unit cost | Ref |
| --- | --- | --- | --- |
| *Consultations* |  |  |  |
| GP visit | Per consultation | £39.23 | [1] |
| Specialist visit | Per consultation | £121.00 | [3] |
| Dermatology visit | Per consultation | £121.00 | [3] |
| Dermatology paediatric visit | Per consultation | £170.00 | [3] |
| A&E visit | Per attendance | £84.29 | [3] |
| *Drugs* |  |  |  |
| Abrocitinib | Per standard pack | £893.76 | [2] |
| Azathioprine | Per standard pack | £2.06 | [2] |
| Baricitinib | Per standard pack | £805.56 | [2] |
| Ciclosporin | Per standard pack | £18.25 | [2] |
| Dupilumab | Per vial (300 mg) | £1,264.89 | [2] |
| Methotrexate | Per standard pack | £2.39 | [2] |
| Mycophenolate mofetil | Per standard pack | £82.26 | [2] |
| Omalizumab | Per vial (150 mg) | £256.15 | [2] |
| Prednisolone | Per standard pack | £0.68 | [2] |
| Tralokinumab | Per vial (150 mg) | £1,070.00 | [2] |
| Upadacitinib | Per standard pack | £805.56 | [2] |

***Legend.*** This table presents the unit costs of various healthcare consultations and medications used in the management of atopic eczema. The costs are referenced from the respective sources listed in the table. 1. [Website. Available:](http://paperpile.com/b/hiIWi1/H9EI) [Service, N.H. National Cost Collection. 2020; Available from: https://www.england.nhs.uk/costing-in-the-nhs/national-cost-collection/.](about:blank) 2. [Website. Available:](http://paperpile.com/b/hiIWi1/3bGN) [Excellence, British National Formulary. 2022; Available from: https://bnf.nice.org.uk/.](about:blank) 3. [Website. Available:](http://paperpile.com/b/hiIWi1/1wwW) [Kent, Personal Social Services Research Unit. 2022; Available from: https://www.pssru.ac.uk/.](about:blank)

***Figure A1.*** Individual trajectories over the initial 12 months follow up on adults.


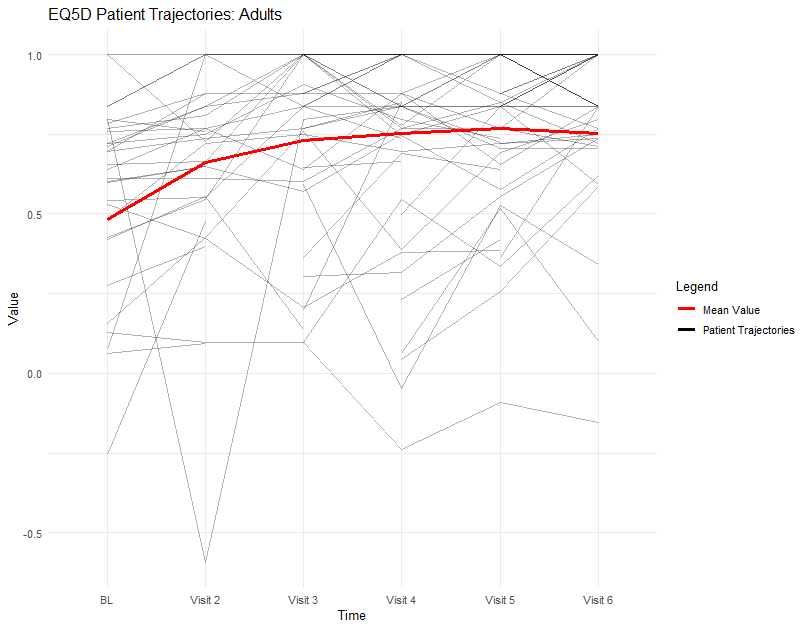


***Figure A2.*** Individual trajectories over the initial 12 months follow up on children.


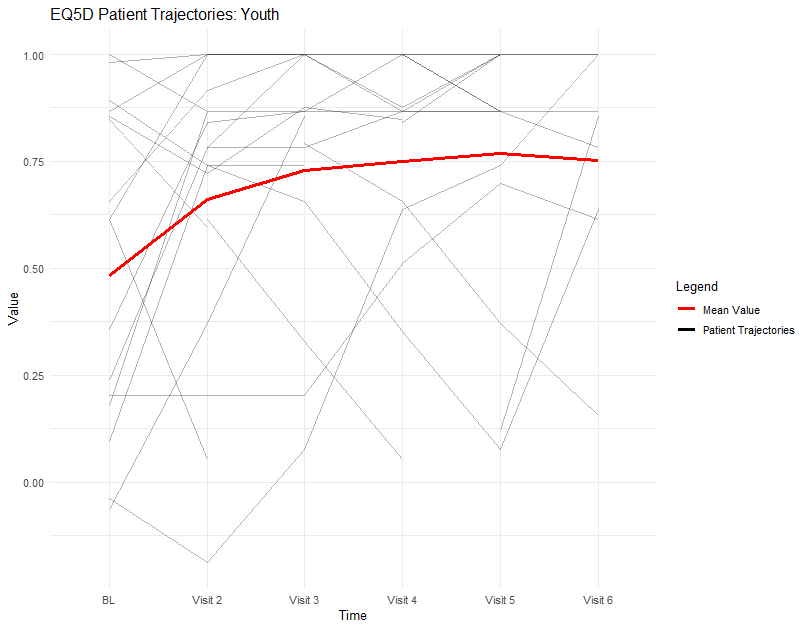


***Figure A3.*** EQ5D dimensions distribution over the initial 12 months follow up on adults.


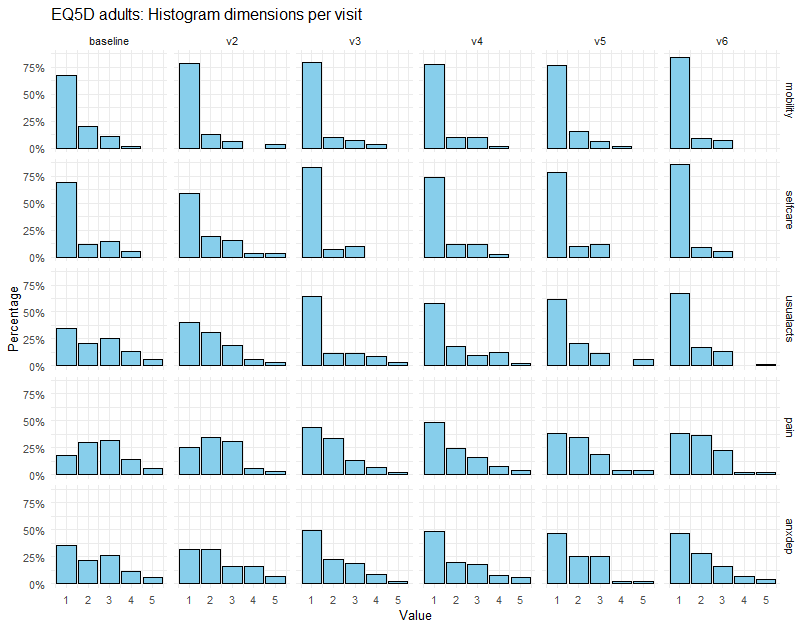


***Figure A4.* EQ5D dimensions distribution over the initial 12 months follow up on children.**

#
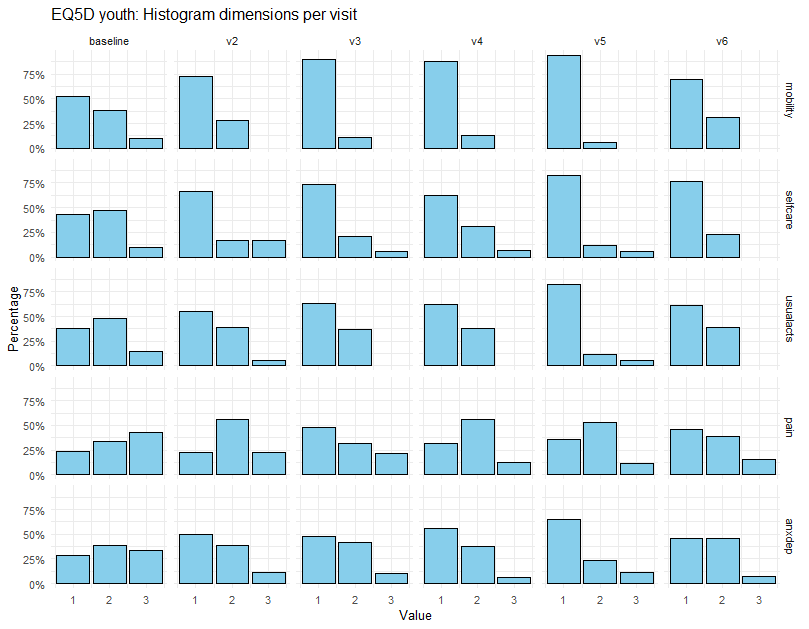


**Table A2. EQ5D scores completeness patterns, by population.**

| ***Adults*** | | | |  | ***Children*** | | | |
| --- | --- | --- | --- | --- | --- | --- | --- | --- |
| ***Freq.*** | ***Percent*** | ***Cum.*** | ***Pattern*** |  | ***Freq.*** | ***Percent*** | ***Cum.*** | ***Pattern*** |
| 14 | 15.05 | 15.05 | 111111 |  | 4 | 16 | 16 | 11111. |
| 7 | 7.53 | 22.58 | 1.1111 |  | 4 | 16 | 32 | 111111 |
| 6 | 6.45 | 29.03 | 1.1.11 |  | 1 | 4 | 36 | .....1 |
| 5 | 5.38 | 34.41 | 1..... |  | 1 | 4 | 40 | ...1.. |
| 5 | 5.38 | 39.78 | 1....1 |  | 1 | 4 | 44 | .111.1 |
| 5 | 5.38 | 45.16 | 1.1... |  | 1 | 4 | 48 | .1111. |
| 4 | 4.3 | 49.46 | 1.1..1 |  | 1 | 4 | 52 | 1..111 |
| 4 | 4.3 | 53.76 | 11.111 |  | 1 | 4 | 56 | 1.1... |
| 4 | 4.3 | 58.06 | 11111 |  | 1 | 4 | 60 | 1.1.11 |
| 39 | 41.94 | 100 | (other patterns) | | 10 | 40 | 100 | (other patterns) |
|  |  |  |  |  |  |  |  |  |

***Legend:*** The table categorizes the frequency and types of data completeness patterns for EQ5D scores among adults and children, providing a cumulative perspective on data completeness in each population group. 1 means that there is a non-missing observation. "." means missing observation. The position of the digit reflects the number of the visit.
